# Supplementary material for: The triple burden of malnutrition among adolescents in Dar es Salaam, Tanzania: The role of gender, household environment, and food insecurity
Source: PLoS One. 2025 Dec 5;20(12):e0337102. doi: 10.1371/journal.pone.0337102 (PMC12680259; doi:10.1371/journal.pone.0337102)

# Adolescent Questionnare

**C0a.Have you read, understood, and signed the consent/assent form?**

- ◯ No
- ◯ Yes

**Interviewer name**

SECTION C: INDIVIDUAL QUESTIONNAIRE ADOLESCENTS 12-19 DEMOGRAPHIC INFORMATION

**Ward**

- Kariakoo
- Mchafukoge
- Ilala
- Vingunguti
- Kipawa
- Kiwalani
- Mnyamani
- Minazi mirefu
- Tabata
- Kinyerezi
- Segerea
- Kimanga
- Liwiti
- Kisukuru
- Ukonga
- Pugu
- Msongola
- Kitunda
- Chanika
- Kivule
- Gongolamboto
- Majohe
- Zingiziwa
- Buyuni
- Pugu Station
- Mzinga
- Kipunguni

**Cluster number**

**Household number**

- HH01
- HH02
- HH03
- HH04
- HH05
- HH06
- HH07
- HH08
- HH09
- HH10
- HH11
- HH12

**Household ID number**

*Household identification ID(Ward letter, cluster number, household number)*

## Demographic information of adolescent (12-19 years)

**C1. Gender**

- Male
- Female

**C2. Date of birth**

yyyy-mm-dd

**C2a. Age (years)**

**C3. Marital status**

- Single
- Cohabit
- Married
- Divorced
- Widowed

**C4. Do you have any children?**

- No
- Yes

**C5. What do you normally do during daytime?**

- School
- Work
- Others

**C5a. It other, mention**

______________________________

**C6. Are you working for a living?**

- No
- Yes

**C7. How are you compensated for your work?**

- Salary
- Own account (payment directly from customer)
- Paid in Kind

**C8. How much do you get paid monthly? (TZ shillings)**

______________________________

**C9. Who oversees your income expenditure?**

- By myself
- Parent/guardian
- Employer
- Sibling/relative
- Others

**C9a. Please specify who has primary control of your income expenditure.**

______________________________

**C10. Have you ever attended school?**

- No
- Yes

**C11. Are you currently attending school?**

- No
- Yes

**C12. What was the last grade you attended before leaving school?**

- Standard 1
- Standard 2
- Standard 3
- Standard 4
- Standard 5
- Standard 6
- Standard 7
- Form I
- Form II
- Form III
- Form IV
- Advanced Secondary
- Technical College

**C13. What is your current grade level?**

- Standard 1
- Standard 2
- Standard 3
- Standard 4
- Standard 5
- Standard 6
- Standard 7
- Form I
- Form II
- Form III
- Form IV
- Advanced Secondary
- Technical College

**C14. What was the main reason for not attending/quitting school?**

- Pregnancy
- Parent decision
- Lack of Tuition/Fees
- Parent's/Guardian's death
- Sickness/permanent disability
- Failed Exams
- Others

**C15. Please mention, if other**

## SECTION D: FOOD FREQUENCY QUESTIONNAIR

SECTION D: FOOD FREQUENCY QUESTIONNAIRE
In this section we are interested in understanding the variety and frequency of foods you've consumed in the past seven(7) days. Please take some time to reflect on your meals and try to recall them as accurately as possible.

**D1. Cassava**

- Never
- Once per week
- 2-4 times a week
- 5-6 times a week
- Everyday
- No answer

**D2. Ugali**

- Never
- Once per week
- 2-4 times a week
- 5-6 times a week
- Everyday
- No answer

**D3. Cooking banana**

- ◯ Never
- ◯ Once per week
- ◯ 2-4 times a week
- ◯ 5-6 times a week
- ◯ Everyday
- ◯ No answer

**D4. Mixed Porridge flour**

- ◯ Never
- ◯ Once per week
- ◯ 2-4 times a week
- ◯ 5-6 times a week
- ◯ Everyday
- ◯ No answer

**D5. Bread roll**

- ◯ Never
- ◯ Once per week
- ◯ 2-4 times a week
- ◯ 5-6 times a week
- ◯ Everyday
- ◯ No answer

**D6. Rice**

- ◯ Never
- ◯ Once per week
- ◯ 2-4 times a week
- ◯ 5-6 times a week
- ◯ Everyday
- ◯ No answer

**D7. Boiled Corn**

- Never
- Once per week
- 2-4 times a week
- 5-6 times a week
- Everyday
- No answer

**D8. Sweet Potatoes**

- Never
- Once per week
- 2-4 times a week
- 5-6 times a week
- Everyday
- No answer

**D9. Potatoes**

- Never
- Once per week
- 2-4 times a week
- 5-6 times a week
- Everyday
- No answer

**D10. Yam**

- Never
- Once per week
- 2-4 times a week
- 5-6 times a week
- Everyday
- No answer

Meat products

**D11. Beef**

- Never
- Once per week
- 2-4 times a week
- 5-6 times a week
- Everyday
- No answer

**D12. Beef Liver**

- Never
- Once per week
- 2-4 times a week
- 5-6 times a week
- Everyday
- No answer

**D13. Goat meat**

- Never
- Once per week
- 2-4 times a week
- 5-6 times a week
- Everyday
- No answer

**D14. Sheep**

- Never
- Once per week
- 2-4 times a week
- 5-6 times a week
- Everyday
- No answer

**D15. Pork**

- Never
- Once per week
- 2-4 times a week
- 5-6 times a week
- Everyday
- No answer

**D16. Rabbit**

- Never
- Once per week
- 2-4 times a week
- 5-6 times a week
- Everyday
- No answer

**D17. Chicken/poultry**

- Never
- Once per week
- 2-4 times a week
- 5-6 times a week
- Everyday
- No answer

**D18. Chicken Liver**

- Never
- Once per week
- 2-4 times a week
- 5-6 times a week
- Everyday
- No answer

**D19. Small Dried Fish**

- Never
- Once per week
- 2-4 times a week
- 5-6 times a week
- Everyday
- No answer

**D20. Fish**

- Never
- Once per week
- 2-4 times a week
- 5-6 times a week
- Everyday
- No answer

**D21. Sea food (street food)**

- Never
- Once per week
- 2-4 times a week
- 5-6 times a week
- Everyday
- No answer

**D22. Other meat/fish**

- Never
- Once per week
- 2-4 times a week
- 5-6 times a week
- Everyday
- No answer

**D23. Milk**

- Never
- Once per week
- 2-4 times a week
- 5-6 times a week
- Everyday
- No answer

**D24. Eggs**

- Never
- Once per week
- 2-4 times a week
- 5-6 times a week
- Everyday
- No answer

Green Leafy Vegetables

**D25. Spinach**

- Never
- Once per week
- 2-4 times a week
- 5-6 times a week
- Everyday
- No answer

**D26. Broccoli**

- Never
- Once per week
- 2-4 times a week
- 5-6 times a week
- Everyday
- No answer

**D27. Pumpkin Leaf**

- Never
- Once per week
- 2-4 times a week
- 5-6 times a week
- Everyday
- No answer

**D28. Hare Lettuce**

- Never
- Once per week
- 2-4 times a week
- 5-6 times a week
- Everyday
- No answer

**D29. Okra**

- Never
- Once per week
- 2-4 times a week
- 5-6 times a week
- Everyday
- No answer

**D30.Taro Leaf**

- Never
- Once per week
- 2-4 times a week
- 5-6 times a week
- Everyday
- No answer

**D31. Other green leafy vegetables**

- Never
- Once per week
- 2-4 times a week
- 5-6 times a week
- Everyday
- No answer

Roots, Cruciferous and Deep Orange Vegetables

**D32. Cabbage**

- Never
- Once per week
- 2-4 times a week
- 5-6 times a week
- Everyday
- No answer

**D33. Butternut**

- Never
- Once per week
- 2-4 times a week
- 5-6 times a week
- Everyday
- No answer

**D34. Carrots**

- Never
- Once per week
- 2-4 times a week
- 5-6 times a week
- Everyday
- No answer

**D35. Beetroot**

- Never
- Once per week
- 2-4 times a week
- 5-6 times a week
- Everyday
- No answer

Fruits

---

**D36. Avocado**

- Never
- Once per week
- 2-4 times a week
- 5-6 times a week
- Everyday
- No answer

**D37. Oranges**

- Never
- Once per week
- 2-4 times a week
- 5-6 times a week
- Everyday
- No answer

**D38. Banana**

- Never
- Once per week
- 2-4 times a week
- 5-6 times a week
- Everyday
- No answer

**D39. Pineapples**

- Never
- Once per week
- 2-4 times a week
- 5-6 times a week
- Everyday
- No answer

**D40. Baobab**

- Never
- Once per week
- 2-4 times a week
- 5-6 times a week
- Everyday
- No answer

**D41. Mango**

- Never
- Once per week
- 2-4 times a week
- 5-6 times a week
- Everyday
- No answer

Legumes

---

**D42. Beans**

- Never
- Once per week
- 2-4 times a week
- 5-6 times a week
- Everyday
- No answer

**D43. Soybean**

- Never
- Once per week
- 2-4 times a week
- 5-6 times a week
- Everyday
- No answer

**D44. Lentils**

- Never
- Once per week
- 2-4 times a week
- 5-6 times a week
- Everyday
- No answer

**D45. Cowpeas**

- Never
- Once per week
- 2-4 times a week
- 5-6 times a week
- Everyday
- No answer

**D46. Chickpeas**

- Never
- Once per week
- 2-4 times a week
- 5-6 times a week
- Everyday
- No answer

**D47. Pigeon peas**

- Never
- Once per week
- 2-4 times a week
- 5-6 times a week
- Everyday
- No answer

**D48. Hyacinth Beans**

- Never
- Once per week
- 2-4 times a week
- 5-6 times a week
- Everyday
- No answer

Discretionary food items

---

**D49. Sweetened Beverages**

- Never
- Once per week
- 2-4 times a week
- 5-6 times a week
- Everyday
- No answer

**D50. Salty Snacks (e.g., samosa, kebab, plantain chips)**

- Never
- Once per week
- 2-4 times a week
- 5-6 times a week
- Everyday
- No answer

**D51. Sweet Snacks (e.g., chocolates, biscuits, cakes, sweets, ice cream)**

- Never
- Once per week
- 2-4 times a week
- 5-6 times a week
- Everyday
- No answer

Fast Food

---

**D52. Pizza**

- Never
- Once per week
- 2-4 times a week
- 5-6 times a week
- Everyday
- No answer

**D53. Burger**

- Never
- Once per week
- 2-4 times a week
- 5-6 times a week
- Everyday
- No answer

**D54. Chips**

- Never
- Once per week
- 2-4 times a week
- 5-6 times a week
- Everyday
- No answer

**D55. Fried Chicken**

- Never
- Once per week
- 2-4 times a week
- 5-6 times a week
- Everyday
- No answer

## SECTION E: SEDENTARY HABITS

Most questions will ask you to think only about the LAST 7 DAYS, but a few questions will ask about what you typically do (during a normal week). THERE ARE NO RIGHT OR WRONG ANSWERS SO PROVIDE HONEST ANSWERS.

**E1. How many days last week did you watch television?**

- Did not watch
- 1 day
- 2 days
- 3 days
- 4 days
- 5 days

**E1a. On the days you watched, how long did you typically watch television each day?**

- Less than one hour
- 1 to 2 hours
- More than two hours

**E2. How many days last week did you play video games, use a smartphone/iPad/computer for leisure activities e.g., social media and schoolwork?**

- Did not watch
- 1 day
- 2 days
- 3 days
- 4 days
- 5 days

**E2a. On the days you did this, how long did you typically spend on these activities each day?**

- Less than one hour
- 1 to 2 hours
- More than two hours

**E3. How many days last week did you spend reading, sitting idly, talking, or playing games (like chess or cards) either alone or with friends/family?**

- ◯ Did not watch
- ◯ 1 day
- ◯ 2 days
- ◯ 3 days
- ◯ 4 days
- ◯ 5 days

**E3a. On the days you did this, how long did you typically spend on these activities each day?**

- ◯ Less than one hour
- ◯ 1 to 2 hours
- ◯ More than two hours

**E4. How many hours a day do you spend doing homework?**

- ◯ I did not do this.
- ◯ Less than one hour
- ◯ 1 to 2 hours
- ◯ More than two hours

On weekends, (Saturday and Sunday)

---

**E5. How long did you spend watching television over the weekend?**

- ◯ I did not do this.
- ◯ Less than one hour
- ◯ 1 to 2 hours
- ◯ More than two hours

**E6. How long did you spend playing video games, using a computer/smartphone/iPad for leisure activities like social media and for schoolwork activities over the weekend?**

- ◯ I did not do this.
- ◯ Less than one hour
- ◯ 1 to 2 hours
- ◯ More than two hours

**E7. How long did you spend reading or just sitting, or playing games (like chess or playing cards) by yourself or with friends or family?**

- I did not do this.
- Less than one hour
- 1 to 2 hours
- More than two hours

**E8. How long did you do homework?**

- I did not do this
- Less than 1 hour
- Between 1 and 2 hours
- More than 2 hours

## SECTION F: PHYSICAL ACTIVITY:

This section asks about physical activities that is, any play, game, sport, exercise at home/ work or school (either during recess or after school), transportation (like walking or cycling to school or work) that gets you moving and breathing harder.

**F1. In the past 7 days, have you play any kind of sport like football, cycling, basketball, skipping, running or participate in physical exercise like dancing, exercise classes or swimming?**

- No
- Yes
- No answer

**F2. In the past 7 days, how many times have you played any sport like football, cycling, basketball, skipping, running or participate in physical exercise , like dancing, exercise classes or swimming**

- 1 day
- 2 days
- 3 days
- 4 days
- 5 days
- 6 days
- 7 days

**F3. In the past 7 days have you walked or cycled to school or work?**

- No
- Yes
- Don't know
- No answer

**F4. How much time have you walked or cycled to reach to school or work? Includes both going to school and returning home.**

- 5 – 15 minutes
- 16 – 30 minutes
- 31 minutes to 1 hour
- More than 1 hour

**F5. On weekdays, when you wake up in the morning while you are at home do you normally do physical activities such as housework, gardening, fetching water etc before/after school for at least 30 minutes?**

- No
- Yes
- Don't know
- No answer

## SECTION G: FOOD ENVIRONMENT

**G1. In the past month in your house, which foods and drinks were frequently available? (Available at least 3-4 days a week)**

*Please select all that apply*

- [ ] Fast foods bought from restaurants e.g.: chicken chips, samosas, kababs, sausages etc.
- [ ] Fresh squeezed juice from fruits (home-made)
- [ ] Sugary drinks such as sodas, artificial/ boxed juices (Azam, Fresh, Ceres, Juice cola etc)
- [ ] Chocolates, sweets
- [ ] Cakes, biscuits
- [ ] Doughnuts, mandazi, chapati
- [ ] Vegetables
- [ ] Fruits
- [ ] Milk and milk products (soured milk, fresh milk, yoghurt)

**G2. Think about the local area around your home; which of the following is easily accessible (a walking distance) ?**

*Please select all that apply.*

- [ ] Street vendors who sell fast foods such as bajia, samosas, chips mayai
- [ ] Markets or local sellers who sell fruits and vegetables.
- [ ] Take away restaurants.

**G3. Over the past one month, have you eaten somewhere else apart from your home?**

- No
- Yes
- No answer

**G4. Where else have you eaten at?**

*Please select all that apply.*

- [ ] School
- [ ] Street vendor/ mama ntilie
- [ ] At a neighbour's/ friend's house
- [ ] At a relative's house (grandparents/ aunt/uncle/siblings)
- [ ] At a fast-food outlet
- [ ] Party
- [ ] Others

**G4a. Please mention all the other places that you eat at**

---

**G5. How many times in a week do you eat out of your home?**

- Once a week
- 2-3 times
- 4-5 times
- 6-7 times
- More than 7 times

**G6. What do you usually eat out of your home?**

*Please select all that apply.*

- [ ] Fruits and vegetables
- [ ] Rice/ ugali/ cassava/ chapati
- [ ] Fast food like fried potatoes, pizzas, burgers, samosas, kebabs, sausages
- [ ] Sugary drinks and snacks like cakes, sweets, sodas, or processed juice
- [ ] Meat or milk.

## SECTION H: HEALTH STATUS

SECTION H: HEALTH STATUS

---

**H1. In general, how do you consider your health?**

- Poor
- Fair
- Good
- Excellent
- No answer

**H2. Do you have any chronic health condition or disability?**

- No
- Yes
- No answer

**H2a. Mention your chronic health condition or disability**

______________________________

**H3. Are you currently attending clinic for the chronic health conditions?**

- No
- Yes
- No answer

**H4. Are you taking any medication?**

- No
- Yes
- No answer

**H5. What is the name of the medication that you are taking?**

______________________________

**H6. Have you ever been diagnosed of any mental illness?**

- No
- Yes
- Don't know
- No answer

**H7. Have you been diagnosed with malaria in the past 6 months or less?**

- No
- Yes
- Don't know
- No answer

**H8. Have you had a fever in the past 7 days?**

- No
- Yes
- Don't know
- No answer

# SECTION I: Health effects and adolescent growth and development (Ask for the past month)

SECTION I: We ask each participant questions about things that can affect their health and well-being. Some questions may not be suitable for you. It's okay to leave some questions open. Please answer these questions on your own, without help from your parent or friends, and be as honest as possible. Your responses are private.

---

**I1. In general, are you happy with the way things are going for you?**

- ◯ Yes
- ◯ Sometimes
- ◯ No
- ◯ Don't know
- ◯ No answer

**I2. Do you get along with your family?**

- ◯ Yes
- ◯ Sometimes
- ◯ No
- ◯ Don't know
- ◯ No answer

**I3. Do you have at least one adult you can really talk to?**

- ◯ Yes
- ◯ Sometimes
- ◯ No
- ◯ Don't know
- ◯ No answer

**I4. Do you ever have a problem of feeling down, depressed, or hopeless?**

- ◯ Yes
- ◯ Sometimes
- ◯ No
- ◯ Don't know
- ◯ No answer

**I5. Do you ever feel anxious or worried for no good reason?**

- Yes
- Sometimes
- No
- Don't know
- No answer

**I6. Do you ever feel bad about yourself or like you are not doing well?**

- Yes
- Sometimes
- No
- Don't know
- No answer

**I7. Do you feel safe at home?**

- Yes
- Sometimes
- No
- Don't know
- No answer

**I8. Do you feel safe at school?**

- Yes
- Sometimes
- No
- Don't know
- No answer

**I9. Do you feel safe in your community?**

- Yes
- Sometimes
- No
- Don't know
- No answer

**I10. Do you think you are about the right weight height?**

- Yes
- Sometimes
- No
- Don't know
- No answer

**I11. Do you ever skip meals, use laxatives, or diet pills, or throw up on purpose to lose weight to control your weight?**

- Yes
- Sometimes
- No
- Don't know
- No answer

## SECTION J: BEHAVIOUR PRACTICES

SECTION J: BEHAVIOUR PRACTICES

**J1. Cigarettes**

- Never
- Rarely(1/2 times)
- Sometimes(a few times)
- Often(Sometimes)
- Always(Almost every day)
- Don't know
- No answer

**J2. Chewing tobacco or snuff, khat**

- Never
- Rarely(1/2 times)
- Sometimes(a few times)
- Often(Sometimes)
- Always(Almost every day)
- Don't know
- No answer

**J3. Local brew, beer**

- Never
- Rarely(1/2 times)
- Sometimes(a few times)
- Often(Sometimes)
- Always(Almost every day)
- Don't know
- No answer

**J4. Imported beer, wine, spirits**

- Never
- Rarely(1/2 times)
- Sometimes(a few times)
- Often(Sometimes)
- Always(Almost every day)
- Don't know
- No answer

**J5. Unprotected sex**

- Never
- Rarely(1/2 times)
- Sometimes(a few times)
- Often(Sometimes)
- Always(Almost every day)
- Don't know
- No answer

## SECTION K: INFORMATION ON ADOLESCENT GIRLS ONLY

SECTION K: INFORMATION ON ADOLESCENT GIRLS ONLY

---

**K1. How many times have you skipped breakfast in the past 7 days?**

- Zero times
- 1 time
- 2 times
- 3 times
- 4 times
- 5 times
- 6 times
- Everyday
- Don't know
- No answer

**K2. How often do you drink tea or coffee?**

- Less than once a day
- 1-2 cups a day
- 3-4 cups a day
- More than 4 cups a day
- Don't know
- No answer

**K3. How often do you consume calcium-rich foods such as milk, cheese, and yogurt?**

- Daily
- 2-3 times a week
- Once a week
- Rarely
- Never
- Don't know
- No answer

**K4. How many times a week do you consume iron rich foods like such as meat, poultry, fish, beans, and leafy greens?**

- Never
- Less than once a week
- 1-2 times a week
- 3-4 times a week
- 5 or more times a week
- Don't know
- No answer

**K5. Are there any foods or food groups that you avoid due to cultural, religious, or personal beliefs or practices?**

- No
- Yes
- Don't know
- No answer

**K5a. Which kind of foods do you avoid due to cultural, religious, or personal beliefs and practices**

**K6. Are there any foods or food groups that you avoid due to influence from friends?**

- No
- Yes
- Don't know
- No answer

**K6a. What kind of foods do you avoid due to influence from friends?**

**K7. Have you begun to menstruate or get you period?**

- No
- Yes
- Don't know
- No answer

**K8. How old were you when you first menstruated or got your period?**

**K9. Do you experience regular menstrual cycles**

- No
- Yes
- Don't know
- No answer

**K10. How would you rate your menstrual flows**

- Heavy Bleeding
- Moderate Bleeding
- Light Bleeding
- No answer

**K11. How many days do you menstruate?**

- Less than 3 days
- 3 – 5 days
- More than 5 days
- No answer

**K12. Do you use any contraceptive pills or injections or medicines?**

- No
- Yes
- No answer

**K13. Have you ever been pregnant?**

- No
- Yes
- No answer

**K13a. What was the outcome of your last pregnancy?**

- Delivery
- Miscarriage
- Abortion

**K14. When did this happen (estimation)?**

- Six(6) months age
- One year ago
- Two years age
- Three or more years age

**K14. What is your baby's date of birth ?**

yyyy-mm-dd

**K15. Are you breastfeeding?**

- No
- Yes
- No answer

**K16. Did you have a caesarean or natural birth**

- Caesarean (C-Section)
- Natural
- Don't know
- No answer

**K17. If so, did you experience any complications during your pregnancy or postpartum period?**

- No
- Yes
- Don't know
- No answer

**K18.What kind of complications?**

*Select all that applies*

- Too much bleeding
- Feeling really tired and weak
- Trouble making milk for baby.
- Felling dizzy or out of breath
- Others
- No answer

**K19. Have you ever had symptoms of anaemia like fatigue, weakness, and pale skin**

- No
- Yes
- Don't know
- No answer

# SECTION L: ANTHROPOMETRIC AND HAEMOGLOBIN MEASUREMENTS

SECTION L: HAEMOGLOBIN AND ANTHROPOMETRIC MEASUREMENTS

---

**Weight 1(kg)**

______________________________

**Weight 2(kg)**

______________________________

**Height 1(cm)**

______________________________

**Height 2(cm)**

______________________________

**Haemoglobin 1 (g/dl)**

______________________________

# Household Identifier

**L2. Ward name**

- Kariakoo
- Mchafukoge
- Ilala
- Vingunguti
- Kipawa
- Kiwalani
- Mnyamani
- Minazi mirefu
- Tabata
- Kinyerezi
- Segerea
- Kimanga
- Liwiti
- Kisukuru
- Ukonga
- Pugu
- Msongola
- Kitunda
- Chanika
- Kivule
- Gongolamboto
- Majohe
- Zingiziwa
- Buyuni
- Pugu Station
- Mzinga
- Kipunungi

**L3. Cluster number**

**L4. Household number**

- ◯ HH01
- ◯ HH02
- ◯ HH03
- ◯ HH04
- ◯ HH05
- ◯ HH06
- ◯ HH07
- ◯ HH08
- ◯ HH09
- ◯ HH10
- ◯ HH11
- ◯ HH12

## SECTION 2: Details of the respondent (Any adult living in the household)

**L6. Household size (all persons who normally live here)**

______________________________

**L7. Are you the head of the household?**

- ◯ No
- ◯ Yes

**L8. How old are you?**

______________________________

**L9. Gender**

- ◯ Male
- ◯ Female

**L10. Marital Status**

- ◯ Single
- ◯ Cohabit
- ◯ Married
- ◯ Divorced
- ◯ Widow/Widower
- ◯ No answer

**L11. Relationship with adolescent boy/girl**

- ◯ Parent
- ◯ Older sibling
- ◯ Relative
- ◯ Adopted/Not a relative
- ◯ Selected adolescent is the head of household
- ◯ Housemaid

**L12. Economic activity of household head**

- ◯ Formal Employment
- ◯ Informal employment
- ◯ Self employed
- ◯ Unemployed
- ◯ Don't Know
- ◯ No answer

**L13. Household head Level of education**

- ◯ No formal education
- ◯ Primary education
- ◯ Secondary Education
- ◯ Diploma/higher education
- ◯ Technical school
- ◯ Don't Know
- ◯ No answer

## Description of the Dwelling

**A1. What is the MAIN material of the walls (in the main living area where the family spends the most time)?**
*Observe the main material and record observation*

- ◯ Brick/cement block
- ◯ Traditional material/mud
- ◯ Wood
- ◯ Corrugated Iron
- ◯ Other

**A1a. If other, mention**

---

**A2. What is the MAIN material of the floors (in the main living room where the family spends the most time)?**

*Observe the main material and record observation*

- ◯ Cement
- ◯ Carpet
- ◯ Dung
- ◯ Normal Wood
- ◯ Sand/Mud
- ◯ Plastic
- ◯ Bamboo
- ◯ Polished wood
- ◯ Tiles
- ◯ Other

**A2a. If other, mention**

______________________________

**A3. What is the main material of the roof (in the main living area where the family spends the most time)?**

*Observe the main roofing material and record observation*

- ◯ No roof
- ◯ Shrubs/Grass
- ◯ Corrugated Iron
- ◯ Mud
- ◯ Bamboo
- ◯ Normal wood
- ◯ Polished wood
- ◯ Cement
- ◯ Makuti
- ◯ Other

**A3a. If other, mention**

______________________________

**A4. What is the primary source of drinking water for your household?**

- ◯ Piped – Inside the house.
- ◯ Piped – Outside but in own yard.
- ◯ Closed well
- ◯ Open well
- ◯ Piped – public tap.
- ◯ River water/dam/lake/pond
- ◯ Tank water
- ◯ Closed Spring surface water
- ◯ Open Spring surface water
- ◯ Borehole
- ◯ Rain water
- ◯ Tanker truck
- ◯ Other

**A4a. If other, mention**

---

**A5. What type of toilet facility does your household typically use?**

- ◯ Flush toilet inside the house
- ◯ Flush toilet outside the house
- ◯ Ventilated pit latrine
- ◯ Pit latrine
- ◯ Bucket toilet
- ◯ Bush/veld / no toilet
- ◯ Other

**A1a. If other, mention**

---

**A6. Is the toilet shared with other households?**

- ◯ No
- ◯ Yes
- ◯ Don't Know
- ◯ No answer

**A7. How many households share the toilet?**

---

**A8. What type of fuel or energy source is most often used for cooking in the household?**

- Electricity
- Gas
- Charcoal
- Wood
- Alcohol/Ethanol
- Gasoline/Diesel
- Kerosene/Paraffin
- Coal/Lignite
- Charcoal
- Agricultural Crop Animal dung / Waste
- Garbage/Plastic
- Other

**A8a. Specify the energy source that is most often used for cooking in the household.**

**A9. How many rooms in this household are used for sleeping?**

**A10. Does any relative send money from abroad?**

- No
- Yes
- Don't Know
- No answer

Does your household have the following items?

**A11. Electricity**

- No
- Yes
- Don't know
- No answer

**A12. Radio**

- No
- Yes
- Don't know
- No answer

**A13. Television**

- No
- Yes
- Don't know
- No answer

**A14. Computer**

- No
- Yes
- Don't know
- No answer

**A15. Telephone (non- mobile)**

- No
- Yes
- Don't know
- No answer

**A16. Refrigerator**

- No
- Yes
- Don't know
- No answer

**A17. Oven**

- No
- Yes
- Don't know
- No answer

**A18. Microwave**

- No
- Yes
- Don't know
- No answer

**A19. Iron**

- No
- Yes
- Don't know
- No answer

**A20. Watch**

- No
- Yes
- Don't know
- No answer

**A21. Mobile telephone**

- No
- Yes
- Don't know
- No answer

**A22. Bicycle**

- No
- Yes
- Don't know
- No answer

**A23. Motorcycle**

- No
- Yes
- Don't know
- No answer

**A24. Car or truck**

- No
- Yes
- Don't know
- No answer

**A25. Bank Account**

- No
- Yes
- Don't know
- No answer

## Household Food Insecurity Access Scale (HFIAS)

**B1. Over the past 30 days, were you concerned that your household might not have sufficient food?**

- No
- Yes
- Don't Know
- No answer

**B1a. How often did this occur?**

- Often (more than ten times in the past 30 days)
- Sometimes (three to ten times in the past 30 days)
- Rarely (once or twice in the past 30 days)

**B2. In the past 30 days, were you or any household members unable to eat preferred foods due to a lack of resources?**

- No
- Yes
- Don't Know
- No answer

**B2a. How often did this occur?**

- Often (more than ten times in the past 30 days)
- Sometimes (three to ten times in the past 30 days)
- Rarely (once or twice in the past 30 days)

**B3. In the past 30 days, were you or any household members forced to eat a limited variety of foods due to a lack of resources?**

- No
- Yes
- Don't Know
- No answer

**B3a. How often did this occur?**

- Often (more than ten times in the past 30 days)
- Sometimes (three to ten times in the past 30 days)
- Rarely (once or twice in the past 30 days)

**B4. In the past 30 days, were you or any household members compelled to eat foods you would rather not have eaten due to a lack of resources for other food types?**

- No
- Yes
- Don't Know
- No answer

**B4a. How often did this occur?**

- Often (more than ten times in the past 30 days)
- Sometimes (three to ten times in the past 30 days)
- Rarely (once or twice in the past 30 days)

**B5. In the past 30 days, were you or any household members obliged to eat smaller meals than needed because there was not enough food?**

- No
- Yes
- Don't Know
- No answer

**B5a. How often did this occur?**

- Often (more than ten times in the past 30 days)
- Sometimes (three to ten times in the past 30 days)
- Rarely (once or twice in the past 30 days)

**B6. In the past 30 days, were you or any household members forced to eat fewer meals in a day due to a lack of food?**

- No
- Yes
- Don't Know
- No answer

**B6a. How often did this occur?**

- Often (more than ten times in the past 30 days)
- Sometimes (three to ten times in the past 30 days)
- Rarely (once or twice in the past 30 days)

**B7. In the past 30 days, was there ever no food to eat of any kind in your household because of lack of resources to get food?**

- No
- Yes
- Don't Know
- No answer

**B7a. How often did this occur?**

- Often (more than ten times in the past 30 days)
- Sometimes (three to ten times in the past 30 days)
- Rarely (once or twice in the past 30 days)

**B8. In the past 30 days, did you or any household member go to sleep at night hungry because there was not enough food?**

- No
- Yes
- Don't Know
- No answer

**B8a. How often did this occur?**

- Often (more than ten times in the past 30 days)
- Sometimes (three to ten times in the past 30 days)
- Rarely (once or twice in the past 30 days)

**B9. In the past 30 days, did you or any household members go an entire day and night without eating anything because there was not enough food?**

- No
- Yes
- Don't Know
- No answer

**B9a. How often did this occur?**

- Often (more than ten times in the past 30 days)
- Sometimes (three to ten times in the past 30 days)
- Rarely (once or twice in the past 30 days)

**L5. Take GPS**

*Allow device to take location*

latitude (x.y °)

longitude (x.y °)

altitude (m)

accuracy (m)

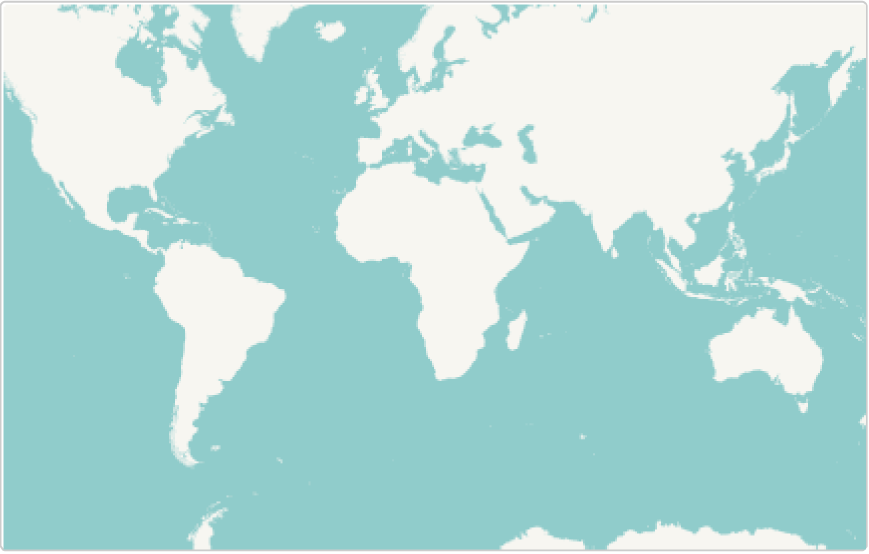

Supplement: S1 File — (PDF) [file pone.0337102.s001.pdf]
