# Supplementary material for: The triple burden of malnutrition among adolescents in Dar es Salaam, Tanzania: The role of gender, household environment, and food insecurity
Source: PLoS One. 2025 Dec 5;20(12):e0337102. doi: 10.1371/journal.pone.0337102 (PMC12680259; doi:10.1371/journal.pone.0337102)
Supplement: Attachment — Submitted filename: Plos One review.docx [file pone.0337102.s003.docx]

The Triple Burden of Malnutrition Among Adolescents in Dar es Salaam, Tanzania: The Role of Gender, Household Environment, and Food Insecurity.

The relevance of the topic in adolescent health fills essential gaps in missing information in LMICs. I’ll encourage the authors to reconsider some points in their document to improve it.

Thank you!

1. Keywords and Framing

Recommendation: Include keywords such as gender, low- and middle-income countries (LMICs), and vulnerable populations to enhance the article’s discoverability and accurately reflect its analytical scope.

Critical Note: The manuscript should adopt an intersectional framework from the outset, positioning gender, socioeconomic deprivation, and adolescent malnutrition as interlinked determinants. This framing should guide the research question, rationale, and interpretation throughout the paper.

2. Introduction

Lines 43–44: Clarify the distinction between physical growth (e.g., linear height, weight gain) and development (e.g., cognitive, emotional, and psychosocial maturation).

Recommendation: Introduce the triple burden of malnutrition—undernutrition, micronutrient deficiencies, and overweight/obesity—early in the text, particularly as it affects adolescents in resource-constrained settings.

Gap: Explicitly address how gender inequities and structural policy shortcomings contribute to adolescent malnutrition in urban, deprived environments. This is essential to contextualize the study's added value.

3. Methodology

a. Study Design

Line 85: Clearly describe the cross-sectional analytical design used. Include references that support the development and validation of the data collection instrument, and describe the steps taken during pre-testing or piloting.

Provide details on enumerator training, field protocols, and quality assurance measures to support claims of internal and external validity.

b. Sampling and Participants

Justify the age range selected, particularly in relation to pregnant and postpartum adolescents, whose nutritional and physiological profiles may differ from non-pregnant peers.

Clarify the sampling frame, cluster selection criteria, and how this sample compares to the general adolescent population in Dar es Salaam.

Avoid fragmentation in the description of inclusion/exclusion criteria and participant characteristics. Reorganize these sections to improve flow and comprehension.

c. Data Collection

Lines 84–89: Clarify distinctions between household-level (family) and individual-level (adolescent) sociodemographic data. If caregiver data contributed to malnutrition and multimorbidity analyses, clearly state this and justify its inclusion.

Articulate the conceptual framework for analyzing the interrelationship between malnutrition indicators (e.g., stunting, thinness, anemia), particularly when these co-occur.

Describe specific controls used to reduce measurement bias from physiological and developmental variability—for example: Adjustment for menstrual cycle timing when measuring anemia in girls;

Use of Tanner staging to contextualize thinness during puberty.

Measurement Tools:

Describe tools used to assess physical activity, referencing validated frameworks (e.g., "Assessment of physical activity among adolescents: a guide to the literature – PMC") and clarify whether this variable was used in the multivariate models.

Detail how wealth index, food insecurity, chronic diseases, disabilities, and mental health were operationalized. Consider disaggregating mental health outcomes if their associations with malnutrition differ.

Define and standardize all covariates used in model adjustments.

For anemia assessment, report on measurement accuracy, precision, tool sensitivity to temperature, and whether altitude adjustments were applied.

Line 154: Correct formatting of bold references.

Justify the decision to collapse food insecurity categories—was this based on statistical power or conceptual reasoning?

Analytical Approach:

Consider a multilevel modeling approach to appropriately account for the hierarchical nature of data (individuals nested within households).

4. Results Interpretation

Recommendation: Reduce the number of tables and concentrate on presenting the most relevant findings. Results should be interpreted through an intersectional lens that considers how gender, socioeconomic conditions, and nutritional outcomes are interrelated.

Provide contextual data: For comparative purposes, cite national or regional statistics on adolescent malnutrition in Dar es Salaam or Tanzania in the introduction or results section.

a. Nutritional Outcomes

Discuss how pubertal development stages (e.g., Tanner stages) influence thinness or overweight prevalence.

Clearly define co-occurrence or coexistence of malnutrition outcomes, including the time frame used to assess simultaneity.

Describe how overlapping forms of malnutrition were analyzed within a physiopathological framework, addressing potential shared or opposing mechanisms.

b. Gender Analysis

Interpret higher prevalence of anemia or thinness in girls through a gendered lens, considering cultural norms, caregiving roles, parasitic exposure, and dietary practices.

Lines 257, 264–277: Consolidate repetitive content regarding causes of anemia to streamline the narrative and improve clarity.

c. Table Improvements

Table 2: Add assumptions and interpretation guidance for Chi-square tests.

Table 6: List the specific chronic illnesses and disabilities reported by participants.

Table 7: Clarify how physical activity levels were cross-tabulated with thinness categories.

Across all tables:

Prioritize clarity and simplicity.

Explain how key covariates were operationalized.

Define how coexisting forms of malnutrition were captured at both individual and household levels.

5. Discussion and Contribution

Structure: The discussion lacks coherence and leans heavily on descriptive repetition. Shift toward interpretive analysis that links results to broader public health and policy contexts.

Avoid speculative explanations for health outcomes (e.g., anemia) without data support.

Clarify the study’s relevance: Why is this research important in Dar es Salaam, and how does it contribute to global efforts to reduce adolescent malnutrition?

Emphasize the study’s contribution to addressing gender and nutrition equity, particularly as a cross-sectional analysis.

Treat gender as a structural determinant of malnutrition, not simply a demographic characteristic—especially given its prominence in the title.

Summarize contributions in terms of:

Filling data gaps on adolescent malnutrition in urban LMICs,

Highlighting gendered vulnerabilities and their implications for targeted interventions.

6. Final Recommendations

Anchor results and discussion within a conceptual framework that integrates gender, socioeconomic status, physiological development, and environmental exposures.

Consider incorporating:

Behavioral models related to diet and physical activity,

Intersectionality frameworks to inform policy recommendations,

Multilevel statistical models to distinguish household and individual effects.

Conclusion: Should synthesize key findings, their relevance to closing equity gaps, and outline practical implications for nutrition and health policy, especially in alignment with Sustainable Development Goals (SDGs).
