# Supplementary material for: The triple burden of malnutrition among adolescents in Dar es Salaam, Tanzania: The role of gender, household environment, and food insecurity
Source: PLoS One. 2025 Dec 5;20(12):e0337102. doi: 10.1371/journal.pone.0337102 (PMC12680259; doi:10.1371/journal.pone.0337102)
Supplement: Attachment — Submitted filename: Response to Reviewers.docx [file pone.0337102.s006.docx]

To the editors: PLOS ONE Journal

Thank you for the valuable feedback provided by the reviewers, which has helped improve the manuscript's quality. Below are detailed responses to each comment, and all revisions have been marked within the text.

**1. Keywords and Framing**

1a. Recommendation: Include keywords such as gender, low- and middle-income countries (LMICs), and vulnerable populations to enhance the article’s discoverability and accurately reflect its analytical scope.

Author's response: We agree that including these keywords will improve the manuscript's visibility and better represent its focus. Accordingly, we have added “gender,” “low- and middle-income countries (LMICs),” and “vulnerable populations**”** to the list of keywords.

1b. Critical Note: The manuscript should adopt an intersectional framework from the outset, positioning gender, socioeconomic deprivation, and adolescent malnutrition as interlinked determinants. This framing should guide the research question, rationale, and interpretation throughout the paper.

Author´s response: Thank you for the comment. We have restructured our manuscript, accordingly, as shown in the 3^rd^ paragraph of the introduction section.

**2. Introduction**

2a. Lines 43–44: Clarify the distinction between physical growth (e.g., linear height, weight gain) and development (e.g., cognitive, emotional, and psychosocial maturation).

Author's response: Thank you for your comment. We have added the distinction in the first paragraph of the introduction lines 44-48

2b. Recommendation: Introduce the triple burden of malnutrition—undernutrition, micronutrient deficiencies, and overweight/obesity—early in the text, particularly as it affects adolescents in resource-constrained settings.

Author's response: We agree that it is important to introduce this concept earlier in the text. We have added the definition of the triple burden of malnutrition in the second paragraph of the introduction from line 50.

2c. Gap: Explicitly address how gender inequities and structural policy shortcomings contribute to adolescent malnutrition in urban, deprived environments. This is essential to contextualise the study's added value.

Author's response: Thank you for pointing this out. We have added this to the manuscript in lines 59-62.

**3. Methodology**

**3a. Study Design**

3a1. Line 85: Clearly describe the cross-sectional analytical design used. Include references that support the development and validation of the data collection instrument, and describe the steps taken during pre-testing or piloting.

Author's response: We have added this information to our revised manuscript in the data collection section In lines 134-136 and 140-142.

3a2. Provide details on enumerator training, field protocols, and quality assurance measures to support claims of internal and external validity.

Author's response: We agree that this is important information; we have added it to the manuscript in lines 131-134 and 156-159.

**3b. Sampling and Participants**

b1. Justify the age range selected, particularly in relation to pregnant and postpartum adolescents, whose nutritional and physiological profiles may differ from non-pregnant peers.

Author's response: Thank you for bringing this important point to our attention. The age range of 12–19 years was selected as it spans the full spectrum of adolescence, from early to late stages, capturing the wide variability in physical development that occurs during this period. We used age-specific measures to assess nutritional status (e.g., weight-for-height Z-scores, age-specific haemoglobin cut-offs), ensuring that developmental differences were appropriately accounted for in the analysis. We excluded pregnant and postpartum adolescents from the study to minimise potential bias, given that their nutritional and physiological needs may differ significantly from those of their non-pregnant peers. This is written in the study population section in lines 106-109.

3b2. Clarify the sampling frame, cluster selection criteria, and how this sample compares to the general adolescent population in Dar es Salaam.

Author's response: Thank you for bringing this to our attention. We have added these details to our revised manuscript, lines 116-120.

3b3. Avoid fragmentation in the description of inclusion/exclusion criteria and participant characteristics. Reorganize these sections to improve flow and comprehension.

Author's response: Thank you for the comment. The method section has been edited to improve the flow.

**3c. Data Collection**

3c1. Lines 84–89: Clarify distinctions between household-level (family) and individual-level (adolescent) sociodemographic data. If caregiver data contributed to malnutrition and multimorbidity analyses, clearly state this and justify its inclusion.

Author's response: Thank you for this valuable comment. We would like to clarify that caregivers only provided information on household-level characteristics, such as household size, parental education, and household assets. They did not respond to questions related to adolescent-level sociodemographic data or health outcomes. All individual-level data, including those used in the analyses of malnutrition and multimorbidity, were obtained directly from the adolescents themselves. We have revised the Methods section to clarify this distinction in lines 141-144.

3c2. Articulate the conceptual framework for analyzing the interrelationship between malnutrition indicators (e.g., stunting, thinness, anemia), particularly when these co-occur.

Author.s response: Thank you for your thoughtful comment. Our analysis was guided by the UNICEF conceptual framework for factors affecting adolescent nutrition, which informed our selection and categorisation of nutritional indicators and explanatory variables. However, we did not conduct an analytical investigation into the interrelationships or shared risk factors among stunting, thinness, and anaemia. Instead, we focused on estimating the burden of each indicator individually, as well as reporting the co-occurrence of two or more forms of malnutrition as a descriptive measure of nutritional multimorbidity. We have added the information on the manuscript lines 160-161

3c3. Describe specific controls used to reduce measurement bias from physiological and developmental variability—for example: Adjustment for menstrual cycle timing when measuring anemia in girls;

Use of Tanner staging to contextualize thinness during puberty.

Author´s response: Thank you for this thoughtful comment. We acknowledge that physiological and developmental factors such as menstrual cycle timing and pubertal stage can influence haemoglobin levels and anthropometric indicators. However, our household survey did not collect data on Tanner staging and menstrual cycle timing, primarily due to the sensitive nature of these topics and the need to minimise respondent burden in a community-based setting, respectively. We have now acknowledged this in the Limitations section lines 379-382 and have recommended that future studies consider including such measures to improve the accuracy of nutritional assessments.

**3d.Measurement Tools:**

3d1. Describe tools used to assess physical activity, referencing validated frameworks (e.g., "Assessment of physical activity among adolescents: a guide to the literature – PMC") and clarify whether this variable was used in the multivariate models.

Author's response: Thank you for this helpful comment. We have clarified in the Methods section that the physical activity questions were adapted from the validated questionnaire used in the International Study of Childhood Obesity, Lifestyle and Environment (ISCOLE) (lines 172-173). Additionally, we have written in the manuscript that all variables were used in multivariate models (line 189-190).

3d2. Detail how wealth index, food insecurity, chronic diseases, disabilities, and mental health were operationalized. Consider disaggregating mental health outcomes if their associations with malnutrition differ.

Author's response: Thank you for your valuable feedback. The variables for chronic diseases and self-rated health were analysed in the same form as they were collected in the questionnaire, without recoding or transformation. Specifically, participants responded to structured questions regarding the presence or absence of chronic diseases and their self-rated health status, and these responses were directly used in the analysis and tabulation. Food insecurity was explained in lines 161-166, and wealth index in lines 194-202.

Regarding mental health, disaggregation was not feasible since only 4 participants reported being diagnosed.

3d3. Define and standardise all covariates used in model adjustments.

Author's response: Thank you for the comment, we have added a table (table 1) with these details under the analysis section.

3d4. For anemia assessment, report on measurement accuracy, precision, tool sensitivity to temperature, and whether altitude adjustments were applied.

Author response: Thank you for your helpful comment. We have revised the Methods section to include additional details on haemoglobin measurement. We used two types of Hemocue devices: Hemocue Hb 201+ and Hemocue Hb 801. Since data collection took place outdoors in Dar es Salaam, we took care to conduct measurements in shaded areas and to limit device exposure to direct sunlight and extreme heat, in accordance with manufacturer guidelines. No altitude adjustments were applied, as Dar es Salaam is located at sea level. These details have been added to the revised Methods section lines 150-156.

3d5. Line 154: Correct formatting of bold references.

Author.s response: Thank you for the comment, we have corrected it.

3d6. Justify the decision to collapse food insecurity categories—was this based on statistical power or conceptual reasoning?

Author's response: The original food insecurity variable had four categories: food secure, mildly food insecure, moderately food insecure, and severely food insecure. For analysis, we collapsed these into two categories (food secure vs. food insecure) due to small sample sizes in some subgroups (eg. the mildly food insecure subgroup), which limited statistical power for detecting meaningful differences across all four levels. We have added the justification to the manuscript lines 168-170.

**3e.Analytical Approach:**

3e1. Consider a multilevel modeling approach to appropriately account for the hierarchical nature of data (individuals nested within households).

**Author's response:** Thank you for the recommendation. However, in our study, we did not use a nested data structure. Instead, we merged household-level data with adolescent-level data and included only one adolescent per household in the final analysis. As a result, there is no clustering of individuals within households, and the data are treated at a single level. Therefore, a multilevel modelling approach was not necessary in this case.

**4. Results Interpretation**

4a **Recommendation**: Reduce the number of tables and concentrate on presenting the most relevant findings. Results should be interpreted through an intersectional lens that considers how gender, socioeconomic conditions, and nutritional outcomes are interrelated.

Author's response: Thank you for the recommendation. We have merged some tables to reduce the number of tables and revised the results interpretation as suggested.

4b **Provide contextual data**: For comparative purposes, cite national or regional statistics on adolescent malnutrition in Dar es Salaam or Tanzania in the introduction or results section.

Author's response: We agree that contextual data are important; we provided data on Dar es Salaam in lines 77-80 in the introduction section of the manuscript.

**4c. Nutritional Outcomes**

4c1. Discuss how pubertal development stages (e.g., Tanner stages) influence thinness or overweight prevalence.

Author's response: Thank you for this valuable comment. Our study did not assess pubertal development stages (e.g., Tanner stages), and therefore we were unable to directly examine their influence on thinness or overweight prevalence in our discussion. We agree that pubertal development may play an important role in adolescent nutritional outcomes, as physiological changes during puberty can affect body composition, growth velocity, and energy requirements. We have now included this point as a recommendation (lines 3798-382) for future research, suggesting that studies incorporate assessments of pubertal stage to better account for developmental differences when examining malnutrition in adolescents.

4c2. Clearly define co-occurrence or coexistence of malnutrition outcomes, including the time frame used to assess simultaneity.

Author's response: Thank you for your comment. In our analysis, co-occurrence (or coexistence) of malnutrition outcomes refers to the presence of two or more forms of malnutrition (e.g., stunting and overweight) in the same individual at the same time. All anthropometric measurements and blood tests used to determine these outcomes were collected during the same time in every individual, and thus reflect a single time point, allowing us to assess simultaneity directly. We have now clarified this in lines 239-240.

4c3 Describe how overlapping forms of malnutrition were analyzed within a physiopathological framework, addressing potential shared or opposing mechanisms.

Authors comment: Thank you for your insightful comment. In our study, due to a smaller sample size, we focused on analysing the individual burden of each form of malnutrition (e.g., anemia, stunting, thinness) separately. We did not examine overlapping or co-existing forms of malnutrition within a physiopathological framework, nor did we analyse shared or opposing mechanisms between them. We acknowledge this as a potential area for future research and have clarified this limitation in the revised manuscript lines (370-371).

**4d. Gender Analysis**

4d1 Interpret higher prevalence of anemia or thinness in girls through a gendered lens, considering cultural norms, caregiving roles, parasitic exposure, and dietary practices.

Author.s response: Thank you for the comment, we have incorporated these points to our revised manuscript.

4d2 Lines 257, 264–277: Consolidate repetitive content regarding causes of anaemia to streamline the narrative and improve clarity.

Author's response: Thank you for bringing this to our attention. We have consolidated the repetitive anameia content to improve narrative and clarity as suggested.

**4e. Table Improvements**

4e1 Table 2: Add assumptions and interpretation guidance for Chi-square tests.

Author's response: We have added the assumption to Table 3 since Table 2 contains only descriptive statistics.

4e2 Table 6: List the specific chronic illnesses and disabilities reported by participants.

Authors response: Thank you for the comment, we have added these details

4e3 Table 7: Clarify how physical activity levels were cross-tabulated with thinness categories.

Authors response: Thank you for the comment, physical activity categories were analysed as they are shown in the table, no further modification to the variables were made.

4e4. Across all tables:

Prioritize clarity and simplicity.

Author´s response: Done

Explain how key covariates were operationalised.

Author´s response: Thank you for the comment. We have explained in the method section lines 177-181

4e5. Define how coexisting forms of malnutrition were captured at both individual and household levels.

Author´s response: Thank you for bringing this to our attention. We have added the information in lines 239-240

**5. Discussion and Contribution**

a. Structure: The discussion lacks coherence and leans heavily on descriptive repetition. Shift toward interpretive analysis that links results to broader public health and policy contexts.

Author´s response: Thank you for the constructive comment. We have revised the discussion toward interpretive analysis and linked to broader public health policy contexts as suggested.

b. Avoid speculative explanations for health outcomes (e.g., anemia) without data support.

Author´s response: Thank you for the comment. We agree that this is important, and we have used reference-backed explanations.

c. Clarify the study’s relevance: Why is this research important in Dar es Salaam, and how does it contribute to global efforts to reduce adolescent malnutrition?

Author s response: Thank you for the comment, we have added this information to our revised discussion lines 363-366.

d.Emphasize the study’s contribution to addressing gender and nutrition equity, particularly as a cross-sectional analysis.

Author´s response: We agree that emphasis on the contribution of the study is important, and we have added the emphasis to the manuscript in lines 359-361.

e. Treat gender as a structural determinant of malnutrition, not simply a demographic characteristic—especially given its prominence in the title.

Author response: Thank you for bringing this to our attention. We have discussed gender as a structural determinant of malnutrition and not a mere biological factor as shown in lines 347-349.

f. Summarise contributions in terms of:

a.Filling data gaps on adolescent malnutrition in urban LMICs,

B. Highlighting gendered vulnerabilities and their implications for targeted interventions.

Author's response: Thank you for the inputs, we have added them to our discussion lines 359-361.

**6. Final Recommendations**

a. Anchor results and discussion within a conceptual framework that integrates gender, socioeconomic status, physiological development, and environmental exposures.

Author s response: Thank you for the recommendation, we have restructured the manuscript to reflect this framework.

b.Consider incorporating:

b.1 Behavioural models related to diet and physical activity,

Author response: Thank you for the valuable suggestion. While we acknowledge the importance of these models in understanding nutrition behaviors, our study primarily focused on identifying the individual and household-level factors associated with malnutrition in adolescents using quantitative survey data. Due to the scope and design of our study, which did not include detailed behavioral or psychosocial measures, we could not incorporate behavioral models in the discussion.

b.2 Intersectionality frameworks to inform policy recommendations,

Author's response: Thank you for the recommendation, we used the intersectionality framework by recommending policies that address multiple factors at once ie gender norms, poverty, sanitation access, in lines 354-358.

b.3 Multilevel statistical models to distinguish household and individual effects.

Author´s response: Thank you for the suggestion. We have responded to this recommendation on the analytical approach section 3e1 above.

**7. Conclusion**: Should synthesize key findings, their relevance to closing equity gaps, and outline practical implications for nutrition and health policy, especially in alignment with Sustainable Development Goals (SDGs).

Thank you for the comment: We have restructured the conclusion as recommended.
