# Supplementary material for: The triple burden of malnutrition among adolescents in Dar es Salaam, Tanzania: The role of gender, household environment, and food insecurity
Source: PLoS One. 2025 Dec 5;20(12):e0337102. doi: 10.1371/journal.pone.0337102 (PMC12680259; doi:10.1371/journal.pone.0337102)
Supplement: Attachment — Submitted filename: REVIEWERS comments - PONE-D-25-13116R1.doc [file pone.0337102.s005.doc]

**REVIEWERS’ QUERIES/COMMENTS/RECOMMENDATIONS FOR MANUSCRIPT PONE-D-25-13116R1**.

**GENERAL COMMENTS/QUERIES/RECOMMENDATIONS**:

1. The manuscript contents pinpoint an important global health concern.
2. The study design and even the quantitative nature of findings provides real-time data for comparison
3. However, there are a number of lexicographic, syntactic as well as logical flaws in almost every section of this manuscript

e.g.: Dar es Salaam city has been judiciously divided into five ‘municipalities’ and not councils as suggested by the authors!

Otherwise, Dar es Salaam city (currently a metropolis proper!) has been evidently reported to have > 5.3 million residents in the most recent Population and Housing Census data conducted in 2022 and reported in 2023. In fact, very recent CIA factsheet estimates approximates the findings to be > 7 million (by July 2024) and hence, it is questionable where did authors get the magic number of 1.6 million?

**SPECIFIC COMMENTS/QUERIES/RECOMMENDATIONS**:

| **Reviewers’ comments/queries/recommendations** | **Section** | **Page number**  **(line number)** | |  |
| --- | --- | --- | --- | --- |
| 1. the fact that there has been no definition of the term ‘*adolescence*’ in the entire manuscript, with authors defining the age group (of 13-19 years) in the study settings sub-section (pp. 9 line 97), can I believe this was the definition of ‘adolescence’ in this study? If so, how different is this definition (based on the specified age group) different from that of ‘teenage’? If not, what was the defining term of the word ‘adolescence’ by the authors??   **Recommendations:** authors are advised to define ‘adolescence’ used to analyse their findings somewhere in the ***introduction section***, in order to avoid unnecessary confusion associated with the blind usage of the term in general literature. | Introduction |  | |  |
| **Reviewers’ comments/queries/recommendations** | | **Section** | **Page number**  **(line number)** | |
| 2.It was not clear what was the ***primary sampling unit*** in this study between ‘households’ or ‘adolescents’? Authors should elaborate it clearly!  **Evidence:** whereas there is evidence of adolescents aged 12-19 years (pp. 9, line 104) as the study population, 12 households in each randomly chosen cluster (pp. 10, line 115), it became apparent that in actual fact, what any given reader would consider to be a total sample size of 507 to be adolescents studied, in fact it was the total number of households!  (refer pp. 16, line 226) | | Methods |  | |
| 3.Why did authors opt for a ‘modified Poisson regression model’ amidst evidence that background studies in the introduction section already suggested the burden of malnutrition (prevalence of anaemia, stunting to be 34%, 16% respectively) among adolescents neither to be ‘*count data*’ nor ‘*rare events*’?  **Reason:** by default, before investigators/authors decide to fit a Poisson regression model if they are dealing with ‘count data’ (which I do not think any of the assessed variables were count data anyways!) as well as justifying assumptions of ‘Poisson regression model’ have been made. | | Methods | 13   (185-186) | |
| 4. Why did authors opt to analyse association between ‘overweight’, ‘thinness’, ‘stunting’ and ‘anaemia’ using Pearson Chi-square test?   **Reason:** Pearson chi-square test is valid in assessing association among ‘*continuous data*’ only. None of the stated variables were ‘*continuous data*’ in the reported manuscript! | | Results | 13  (184 - 185) | |
| 5. Why didn’t authors also include a test for *interation*(s) (effect modification) in their final analysis if there seems to be evidence of *a possible statistically significant interaction* between the factors ‘*overweight*’ and ‘*thinness*’?   **Recommendations:** Authors are advised to consult ‘a chartered statistician’ or ‘qualified biostatistician’ for assistance on the matter! | | Results | 19  (234- 236) | |
