# Supplementary material for: The triple burden of malnutrition among adolescents in Dar es Salaam, Tanzania: The role of gender, household environment, and food insecurity
Source: PLoS One. 2025 Dec 5;20(12):e0337102. doi: 10.1371/journal.pone.0337102 (PMC12680259; doi:10.1371/journal.pone.0337102)
Supplement: Attachment — Submitted filename: Response_to_reviewers_auresp_2.docx [file pone.0337102.s007.docx]

To the Editors of *PLOS ONE*,

We sincerely appreciate the insightful feedback from the reviewers, which has greatly contributed to enhancing the quality of our manuscript. Presented below are our detailed responses to each comment, and all corresponding revisions have been clearly indicated in the text.

**GENERAL COMMENTS/QUERIES/RECOMMENDATIONS**:

1. The manuscript contents pinpoint an important global health concern.
2. The study design and even the quantitative nature of findings provides real-time data for comparison
3. However, there are a number of lexicographic, syntactic as well as logical flaws in almost every section of this manuscript

e.g.: Dar es Salaam city has been judiciously divided into five ‘municipalities’ and not councils as suggested by the authors!

Otherwise, Dar es Salaam city (currently a metropolis proper!) has been evidently reported to have > 5.3 million residents in the most recent Population and Housing Census data conducted in 2022 and reported in 2023. In fact, very recent CIA factsheet estimates approximates the findings to be > 7 million (by July 2024) and hence, it is questionable where did authors get the magic number of 1.6 million?

**AUTHORS' RESPONSE**: We thank the reviewer for this insightful comment. We would like to clarify that in this study, the term councils refers to the five administrative units within Dar es Salaam Region Dar es Salaam City Council (formerly Ilala Municipal Council), Kinondoni, Temeke, Ubungo, and Kigamboni—which are officially recognised as municipal councils under Tanzania’s local government structure.

Regarding the population figure, the number of 1.6 million refers specifically to the **Dar es Salaam City Council (Ilala Municipality)**, where the study was conducted, rather than the entire Dar es Salaam Region. According to the **2022 Population and Housing Census (National Bureau of Statistics, reported in 2024)**, the Dar es Salaam Region indeed has a population exceeding 5.3 million. However, the population of the Dar es Salaam City Council (Ilala Municipality) is reported as approximately **1.6 million** in the same source (NBS, 2024; [link to the census data](https://sensa.nbs.go.tz/publication/Dar.pdf)).

**SPECIFIC COMMENTS/QUERIES/RECOMMENDATIONS**:

| **Reviewers’ comments/queries/recommendations** | **Section** | **Page number**  **(line number)** | |
| --- | --- | --- | --- |
| 1. the fact that there has been no definition of the term ‘*adolescence*’ in the entire manuscript, with authors defining the age group (of 13-19 years) in the study settings sub-section (pp. 9 line 97), can I believe this was the definition of ‘adolescence’ in this study? If so, how different is this definition (based on the specified age group) different from that of ‘teenage’? If not, what was the defining term of the word ‘adolescence’ by the authors??   **Recommendations:** authors are advised to define ‘adolescence’ used to analyse their findings somewhere in the ***introduction section***, in order to avoid unnecessary confusion associated with the blind usage of the term in general literature.  **AUTHORS' RESPONSE**: We thank the reviewer for this thoughtful comment. In our study, we defined adolescence as individuals aged 12–19 years (as shown in the study population section). While the World Health Organisation defines adolescence as the period between 10 and 19 years, we excluded those aged 10–11 years because studies show that adolescents from the age of 12 may have the capacity to be decision-making competent, hence likely to make an informed participation compared to younger ones. We have now clarified this and defined adolescence explicitly in the introduction lines 49-52 to avoid ambiguity, and **have added a reference to back up our age selection.** | Introduction |  | |
| **Reviewers’ comments/queries/recommendations** | | **Section** | **Page number**  **(line number)** |
| 2.It was not clear what was the ***primary sampling unit*** in this study between ‘households’ or ‘adolescents’? Authors should elaborate it clearly!  **Evidence:** whereas there is evidence of adolescents aged 12-19 years (pp. 9, line 104) as the study population, 12 households in each randomly chosen cluster (pp. 10, line 115), it became apparent that in actual fact, what any given reader would consider to be a total sample size of 507 to be adolescents studied, in fact it was the total number of households!  (refer pp. 16, line 226)  **AUTHORS' RESPONSE**: We thank the reviewer for this important comment. To clarify, our study employed a **multi-stage cluster sampling design**, in which **clusters were the first-stage primary sampling units (PSUs)**. Within each selected cluster, 12 households were randomly selected, and from each household, **one eligible adolescent aged 12–19 years** was randomly chosen to participate, resulting in a total sample of 507 adolescents. We have reviewed the methods section to make it clear (lines 120-124). | | Methods |  |
| 3.Why did authors opt for a ‘modified Poisson regression model’ amidst evidence that background studies in the introduction section already suggested the burden of malnutrition (prevalence of anaemia, stunting to be 34%, 16% respectively) among adolescents neither to be ‘*count data*’ nor ‘*rare events*’?  **Reason:** by default, before investigators/authors decide to fit a Poisson regression model if they are dealing with ‘count data’ (which I do not think any of the assessed variables were count data anyways!) as well as justifying assumptions of ‘Poisson regression model’ have been made.  **AUTHORS' RESPONSE:** We thank the reviewer for this insightful comment and the opportunity to clarify our choice of statistical model. We acknowledge that a standard Poisson regression is typically used for count data. However, in this study, we employed a modified Poisson regression model with robust error variance, which is appropriate for binary outcome variables in cross-sectional studies when the outcome is not rare.  The modified Poisson approach was used to directly estimate prevalence ratios (PRs) rather than odds ratios (ORs), which can overestimate associations when outcomes are common (prevalence >10%).  Although the study outcomes (e.g., anaemia, stunting, thinness, overweight/obesity) were binary rather than traditional count data, they can be conceptualised as **counts within a contingency table** (e.g., number of adolescents with or without anaemia by gender or food insecurity status). In this context, **Poisson log-linear models** are theoretically appropriate for modelling such data, which further supports the validity of using a modified Poisson regression with robust variance in this study. | | Methods | 13   (185-186) |
| 4. Why did authors opt to analyse association between ‘overweight’, ‘thinness’, ‘stunting’ and ‘anaemia’ using Pearson Chi-square test?   **Reason:** Pearson chi-square test is valid in assessing association among ‘*continuous data*’ only. None of the stated variables were ‘*continuous data*’ in the reported manuscript!  **AUTHORS' RESPONSE:** We thank the reviewer for their comment. We would like to clarify that the Pearson Chi-square test is appropriate for assessing associations between categorical variables, such as overweight, thinness, stunting, and anaemia in our study. The reviewer may have confused the Pearson Chi-square test with the Pearson product-moment correlation, which is used for continuous variables. In contrast, the Chi-square test compares observed versus expected frequencies in a contingency table and is standard practice for categorical data. | | Results | 13  (184 - 185) |
| 5. Why didn’t authors also include a test for *interation*(s) (effect modification) in their final analysis if there seems to be evidence of *a possible statistically significant interaction* between the factors ‘*overweight*’ and ‘*thinness*’?   **Recommendations:** Authors are advised to consult ‘a chartered statistician’ or ‘qualified biostatistician’ for assistance on the matter!  **AUTHORS' RESPONSE:** We thank the reviewer for this insightful comment regarding potential interaction (effect modification) between overweight and thinness. In our analysis, overweight and thinness were treated as mutually exclusive categorical outcomes, meaning that a child could not simultaneously be both overweight and thin. Therefore, statistical interaction between these two variables is not applicable in this context. However, we agree that assessing interactions is important in general. | | Results | 19  (234- 236) |
